# Regional Differences in the Gut Microbiota and Gut-Associated Immunologic Factors in the Ileum and Cecum of Rats With Collagen-Induced Arthritis

**DOI:** 10.3389/fphar.2020.587534

**Published:** 2020-11-24

**Authors:** Huihui Xu, Jinfeng Cao, Xiaoya Li, Xiangchen Lu, Ya Xia, Danping Fan, Hongyan Zhao, Dahong Ju, Cheng Xiao

**Affiliations:** ^1^ Beijing Key Laboratory of Research of Chinese Medicine on Prevention and Treatment for Major Diseases, Experimental Research Center, China Academy of Chinese Medical Science, Beijing, China; ^2^ Institute of Clinical Medicine, China-Japan Friendship Hospital, Beijing, China; ^3^ The Institute of Medicinal Plant Development, Chinese Academy of Medical Sciences/Peking Union Medical College, Beijing, China; ^4^ School of Traditional Chinese Medicine, Beijing University of Chinese Medicine, Beijing, China; ^5^ Department of Emergency, China-Japan Friendship Hospital, Beijing, China

**Keywords:** gut microbiota, intestinal mucosal immunity, rheumatoid arthritis, ileum, cecum, collagen-induced arthritis

## Abstract

Rheumatoid arthritis (RA) is a common autoimmune disease characterized by chronic inflammation and a multifactorial etiology. We previously showed that gut microbiota dysbiosis in the rat ileum is involved in the development of collagen-induced arthritis (CIA). The gut microbiota in the distinct gastrointestinal tract (GIT) plays region-specific roles, but information on the different roles of the microbiota in distinct GIT compartments of CIA rats is limited. This study aimed to evaluate the region-specific differences in the gut microbial communities and certain gut-associated immunologic factors in the ileum and cecum of CIA rats. Ileal and cecal digesta were collected from CIA and control rats for microbiome analysis. We determined the microbial richness, diversity and taxa as well as the expression of interleukin (IL)-1β and IL-17A in the epithelium and lamina propria of the ileum and cecum mucosal layers. The CIA-induced microbiota alterations in the ileum differed from those in the cecum. The ileal microbiota were more markedly influenced in CIA, as revealed by sharp reductions in the abundances of the families Enterococcaceae, Lactobacillaceae and Streptococcaceae and the genera *Lactobacillus* and *Lactococcus.* Moreover, significant increases in IL-1β, and IL-17A mRNA expression were detected in only the ileal epithelium and lamina propria of the mucosal layer. Therefore, the microbial characteristics in the ileum were consistent with the immune-mediated inflammatory features of CIA, suggesting that the ileal microbiota might better represent the CIA-induced inflammatory responses than the cecal microbiota and that these responses might partially impact the progression of RA by regulating intestinal mucosal immunity.

## Introduction

Rheumatoid arthritis (RA), one of the most frequently occurring autoimmune diseases, is characterized by synovial inflammation, joint cartilage and bone destruction. The onset and progression of RA is believed to require both genetic and environmental factors (Forbes et al., 2016). The role of the gut microbiota in the occurrence and development of RA is increasingly being appreciated. Recently, numerous studies have shown that dysbiosis of the gut microbiota community might function as a crucial environmental factor that triggers the pathogenesis of RA (Liu et al., 2013). The gut microbiota plays a pivotal role in the maintenance of immune system homeostasis, particularly the barrier function of the intestinal mucosa, and an imbalanced interaction between the gut microbiota and the host intestinal mucosal immune system can increase the risk of immune-mediated inflammatory disease (Maynard et al., 2012; Honda and Littman, 2016). We previously found that the intestinal immune response is actively involved in the pathogenesis of collagen-induced arthritis (CIA) in rats, and Peyer’s patch (PP) cells, which are an important component of the intestinal mucosal immune system, could induce immune tolerance to enhance CIA treatment (Xiao et al., 2009). The balance between the immune response and immunologic tolerance in the intestinal tract is important for the maintenance of homeostasis. Bagchi et al. showed that the intestinal mucosal immune response is enhanced in CIA rats, showing increased ratios of CD4^+^/CD8^+^ cells in the epithelium and lamina propria of the small intestine (Wang et al., 2015). Gut-associated lymphoid tissue, which consists of PPs, intestinal intraepithelial lymphocytes (IELs), lamina propria lymphocytes (LPLs) and mesenteric lymph nodes, is the largest lymphoid organ of the human body and is crucial for human health (Chandran et al., 2003), and PPs and other immunocytes of considerable proportions are mainly distributed along the ileum (Ishii et al., 2010).

The gastrointestinal tract (GIT) of vertebrates is in contact with numerous commensal microbiota and exogenous antigens, and the microbial communities are integral to the maintenance of intestinal morphology and nutrient digestion and metabolism and are critical modulators of host immune homeostasis (Donaldson et al., 2018; Mao et al., 2018). The distinct components of the intestine should be regarded as separate entities due to their different regulatory properties in mucosal immunity (Mann et al., 2016). We previously found obvious differences in CD4^+^ and CD8^+^ T cell alterations in PPs, IELs and LPLs between normal and CIA mice, which suggests that the correlations between enteric immune responses and CIA in diverse compartments might differ (Xiao et al., 2006). Different locations in the GIT provide different nutrient and physicochemical conditions for the gut microbiota community. A previous study suggested that the functional heterogeneity of each GIT niche gives rise to regional-specific differences in the gut microbiota (Martinez-Guryn et al., 2019). Our previous study showed that the ileal microbial community of rats with CIA differed from that of the control group (Li et al., 2020). The ileum mainly functions to digest food, absorb nutrients, and develop intestinal mucosal immunity to protect against pathogens (Williams et al., 2015). The cecum is an important site of water and electrolyte absorption, digesta retention and microbial fermentation. The ileum and cecum, which are part of the small and large intestines, respectively, have distinct structures, physiological functions and bacterial loads. Therefore, these areas might exhibit striking differences in their bacterial colonization and related immunologic factor modulation in RA. Because the CIA model shares many clinical, histopathological and immunological features with RA patients, it is always used to investigate novel approaches for the prevention of RA (Zhao et al., 2018). However, only a few previous CIA-related experimental studies have focused on distinguishing gut microbiota dysbiosis in the ileum from that in other intestinal compartments, and information regarding the correlation of the gut microbiota in distinct compartments with mucosal immune responses is scarce. Interleukin (IL)-1β and IL-17A are important inflammatory cytokines implicated in the pathogenesis of RA, and blockage of these molecules alleviates the severity of CIA in mice (Wu et al., 2016). Moreover, some anti-IL-17A monoclonal antibodies could improve RA signs and symptoms in RA patients, and no strong adverse safety signals were noted (Genovese et al., 2010; Genovese et al., 2014). In this study, we evaluated alterations in the gut microbial communities in the ileum and cecum of CIA rats via the high-throughput sequencing of bacterial 16S rRNA and compared the mRNA protein expression of IL-1β and IL-17A in the ileum and cecum of CIA rats.

## Materials and Methods

### Animals

Eighteen adult male Sprague-Dawley rats (190 ± 10 g) were all obtained from the Research Institute of Experimental Animals, Chinese Academy of Medical Science (animal license number: SCXK (Beijing) 2014-0013). The rats were maintained under specific pathogen-free conditions in a conventional animal housing facility at the experimental animal center of China-Japan Friendship Hospital (experimental animal room license number: SCXK (Beijing) 2016-0043) under a 12-h light/12-h dark cycle with 45–65% humidity and a temperature of 20–22°C. Rats were housed in 545 × 395 × 200 mm cages with a maximum of five animals per cage and given free access to the same standard diet and sterile water. Rats were acclimated and cohoused for a 1 week period before the experiments started.

### Induction of Collagen-Induced Arthritis and Animal Grouping

The rats were randomly divided into two groups, the control group (n = 8) and the CIA group (n = 10). Arthritis was induced through an intradermal tail vein injection of 100 μg of bovine type II collagen (Chondrex) in a 1:1 emulsion mixed with an equal amount of incomplete Freund’s adjuvant (Chondrex). Seven days later, a booster injection of the same preparation was administered at the base of the tail. Every 3 days after the booster immunization, the rats were assessed for arthritis severity according to the arthritis index (AI). The AI scores, which range from 0 to 4, are defined as follows: 0) normal; 1) detectable arthritis with slight erythema; 2) significant erythema and swelling; 3) severe erythema plus edema from joint to digit; and 4) maximal edema with arthrolysis. The total arthritis score was then calculated as the sum of the scores of each hind paw. On the 10th day after the primary immunization, the ankle joints were not swollen in one rat, which was excluded. Animals in both the normal and CIA groups were given the same deionized water and standard diet for another 4 weeks.

### Bacterial DNA Extraction, Amplification and Sequencing

At the end of the experiment (38 days after the primary immunization), the rats were sacrificed via anesthesia, and the intestinal contents of the ileum and cecum were harvested, immediately shock-frozen in liquid nitrogen and stored at −80°C for further analysis. Microbial genomic DNA from the ileal and cecal digesta samples was extracted using a QIAamp DNA Stool Mini Kit (Qiagen, Hilden, Germany) according to the manufacturer’s instructions. The quantity and quality of extracted DNAs were measured using a NanoDrop instrument and FastQC software. The PCR products were purified by silane magnetic beads of an appropriate size, and the purified amplicons were sequenced using a Qubit fluorometer (version 2.0, Invitrogen) according to standard protocols. The V3-V4 region of the bacterial 16S rRNA gene was amplified by PCR using the primers 341F and 805R in 25-μL reaction volumes, which included 0.15 μl of 2G Robust DNA polymerase (5 U/μl; KAPA). The PCR thermal cycle was 95°C for 5 min followed by 25 cycles of 95°C for 30 s, annealing at 55°C for 30 s, 72°C for 60 s and a final extension at 72°C for 10 min.

### High-Throughput Sequencing and Microbiome Analysis

We mainly used Quantitative Insights Into Microbial Ecology (QIIME) software for the quality-based filtering of the raw sequences. The universal primer pair was designed to target highly conserved regions of bacterial DNA, and high-throughput sequencing of bacterial 16S rRNA gene marker amplicons encoding the V3 and V4 regions was performed on the Illumina HiSeq 2500 PE250 platform by BioMiao Biological Technology (Beijing) Co., Ltd. The paired-end reads were detected by HTQC (version 1.92.3) to remove ambiguous base sequences and merged into a complete read; the chimeric sequences were identified and removed using Mothur (version 1.38.1).

After the detection of chimeras, the remaining high-quality sequences were analyzed and clustered into operational taxonomic units (OTUs) based on a nucleic acid similarity cutoff of 97% using the QIIME software package and the Usearch612 pipeline. The OTUs were then annotated using the Ribosomal Database Project classifier to obtain the taxonomic assignments, and the Python Nearest Alignment Space Termination method was adopted to align the representative sequences against the Greengenes 16S rDNA database. The relative abundances (%) were estimated at the phylum, class, order, family and genus levels. The data were then processed to calculate the *α*- and *β*-diversity and thus assess the differences between the groups. The *α*-diversity analysis (including the Shannon, Simpson, Chao1, and observed species indices) and the ß-diversity analysis (UniFrac distance) were performed using QIIME.

### Real-Time PCR

Partial gut tissue samples of the ileum and cecum were collected from rats of each group and processed on ice immediately after dissection. The ileum and cecum were opened longitudinally, washed three times with saline and stored at −80°C. The relative mRNA expression levels of IL-1β and IL-17A in the tissue homogenates obtained from the ileum and cecum were determined by real-time quantitative PCR, and GAPDH was used as an endogenous control. Briefly, total RNA was extracted using TRIzol reagent (Invitrogen), and the obtained RNA was used to generate cDNA using a Reverse Transcription System (Promega) according to the manufacturer’s directions. Real-time quantitative PCR was performed using 2 × SYBR Green qPCR Mix (Aidlab Biotechnologies Co., Ltd.). Denaturation was performed at 95°C for 2 min, followed by 40 cycles of 95°C for 15 s and 60°C for 30 s. The following primers were used: IL-1β, forward ACA​GCA​GCA​TCT​CGA​CAA​GAG​C and reverse CCA​CGG​GCA​AGA​CAT​AGG​TAG​C, and IL-17A forward TGT​GTC​AAT​GCG​GAG​GGA​AAG​C and reverse CAC​ACC​CAC​CAG​CAT​CTT​CTC​G. The relative gene expression levels were calculated by the 2^−△△Ct^ method.

### Immunohistochemistry

Partial tissue samples of the ileum and cecum were harvested after the contents were collected, flushed with physiological saline, dissected longitudinally and fixed in formaldehyde. The tissues were subsequently embedded in paraffin, and sections of 4 μm thickness were cut from the paraffin-embedded tissues. IL-1β, and IL-17A were immunolocalized in tissues according to the manufacturer’s instructions. The paraffin sections were dewaxed using routine methods, incubated with 3% H_2_O_2_ for 10 min and treated with primary antibodies against rat IL-1β (Santa Cruz Biotechnology) and IL-17A (Abcam, Cambridge, MA, United States) at 37°C for 1 h. The sections were then incubated with poly-HRP anti-rabbit IgG for 10 min at room temperature, stained with 3,3-diaminobenzidine (Fuzhou Maixin Biotech. Co., Ltd.) and counterstained with hematoxylin.

### Statistical Analysis

The statistical analyses were performed using SPSS 20.0 software. The normality of variable distribution was analyzed using the Shapiro-Wilk test. The microbial diversity data that exhibited a normal distribution were assessed using the independent-sample t-test procedure. The Mann-Whitney test was used to assess the variables with a non-normal distribution. Data were expressed as means ± SEMs. Differences were considered significant if *p* ≤ 0.05. The ß-diversity was visualized using principal coordinate analysis (PCoA) and nonmetric multidimensional scaling (NMDS). The OTU relative abundance values were analyzed using the linear discriminant analysis effect size (LEfSe) algorithm to identify taxa, and the effect size of each differentially abundant feature was estimated by linear discriminant analysis.

## Results

### Collagen-Induced Arthritis Rats Exhibit Different Diversities and Community Structures in the Ileum and Cecum

**FIGURE 1:**
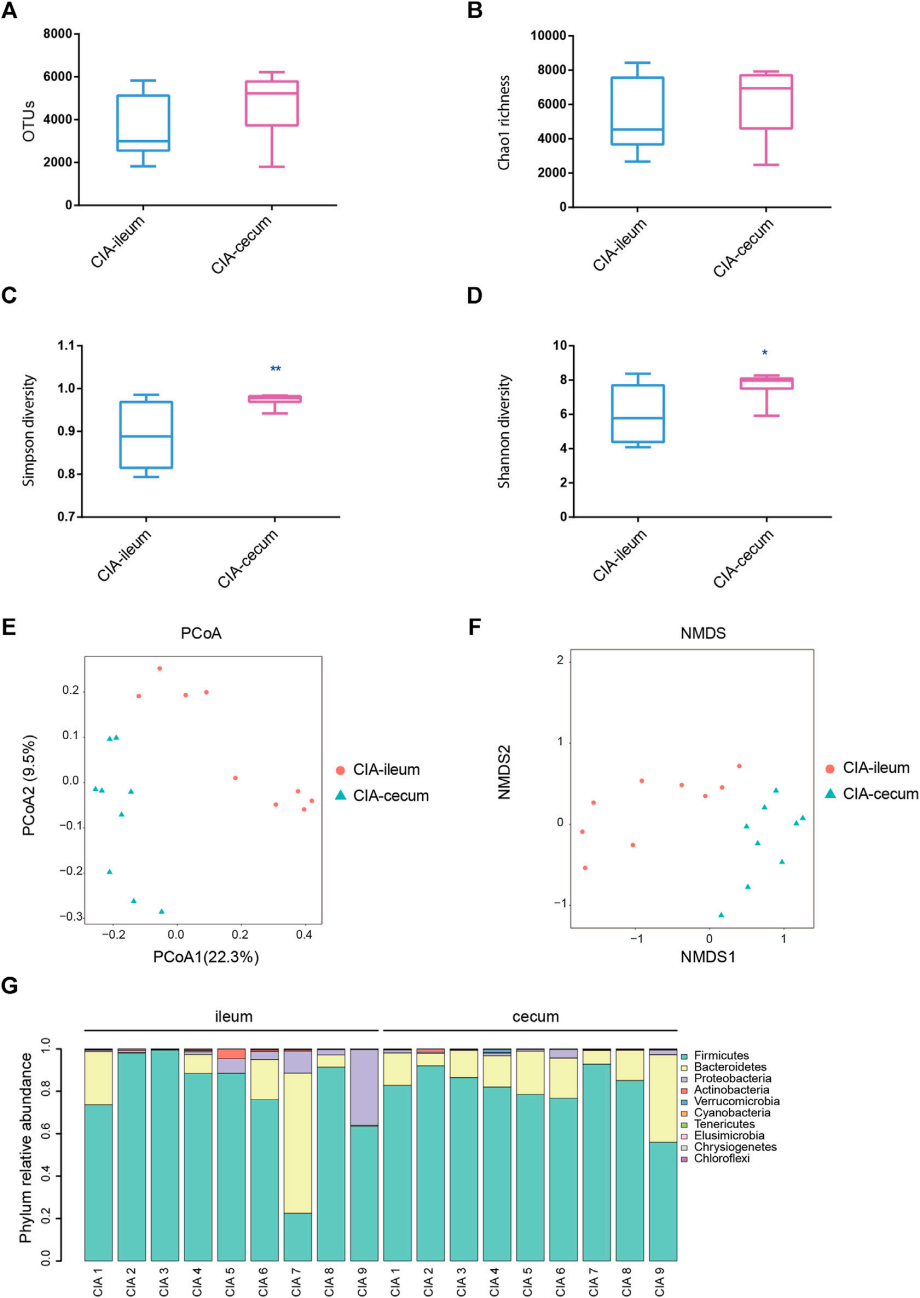
The microbial communities in the ileum and cecum of the collagen-induced arthritis (CIA) group were obtained by 16S sequencing. The rats were induced with bovine collagen II on days 0 and 7 and sacrificed via anesthesia 6 weeks after the primary immunization. **(A)** Analysis of the observed species in the ileal and cecal microbial communities. **(B)** Analysis of the Chao richness estimators of the ileal and cecal microbial communities. **(C)** Analysis of the Simpson diversity indices of the ileal and cecal microbial communities. **(D)** Analysis of the Shannon diversity indices of the ileal and cecal microbial communities. **(E)** Community structure as determined by principal coordinate analysis. **(F)** Nonmetric multidimensional scaling ordination of the microbiome. **(G)** Phylum relative abundances of the microbiota in each sample of CIA rats. The values are presented as the means ± SDs. *p < 0.05 and **p < 0.01 compared with the ileum.

Significant differences in the severity of ankle joint swelling were found between the control and CIA groups (Supplementary Figure S1). A total of 3,346,939 V3-V4 16S rRNA sequence reads were obtained from 17 samples (control group n = 8; CIA group n = 9). Although no significant differences in the OTUs or Chao1 richness estimators were found between the ileum and cecum in the control groups, the ileum and cecum of healthy rats showed obvious differences in bacterial diversity (Supplementary Figure S2). Following CIA treatment, the rats displayed significant differences in the microbial richness values in the ileum and cecum, as demonstrated by the Shannon and Simpson indices (Figures 1C,D). No significant differences in the OTUs or Chao richness estimators were found between the ileum and cecum in the CIA groups (Supplementary Figures S1A,B). As shown in Figure 1E, the PCoA results based on ß-diversity revealed separation of the microbial community in the ileum from that in the cecum, and the divergence in the distribution of the microbiota was also significant (ANOSIM, R = 0.578, *p <* 0.01). Additionally, similar visual separation was achieved by NMDS ordination (Figure 1F). In summary, the ileal and cecal communities of the CIA group showed obvious differences in bacterial diversity and community. After CIA treatment, Firmicutes (77.99%), Bacteroidetes (13.93%) and Proteobacteria (6.99%) were the three predominant phyla in the ileum, and based on their relative abundances, Firmicutes (81.41%) and Bacteroidetes (16.67%) remained the two predominant phyla in the cecum (Figure 1G).

### Collagen-Induced Arthritis Rats Exhibit Alterations in Ileal and Cecal Microbial Richness and Composition

**FIGURE 2:**
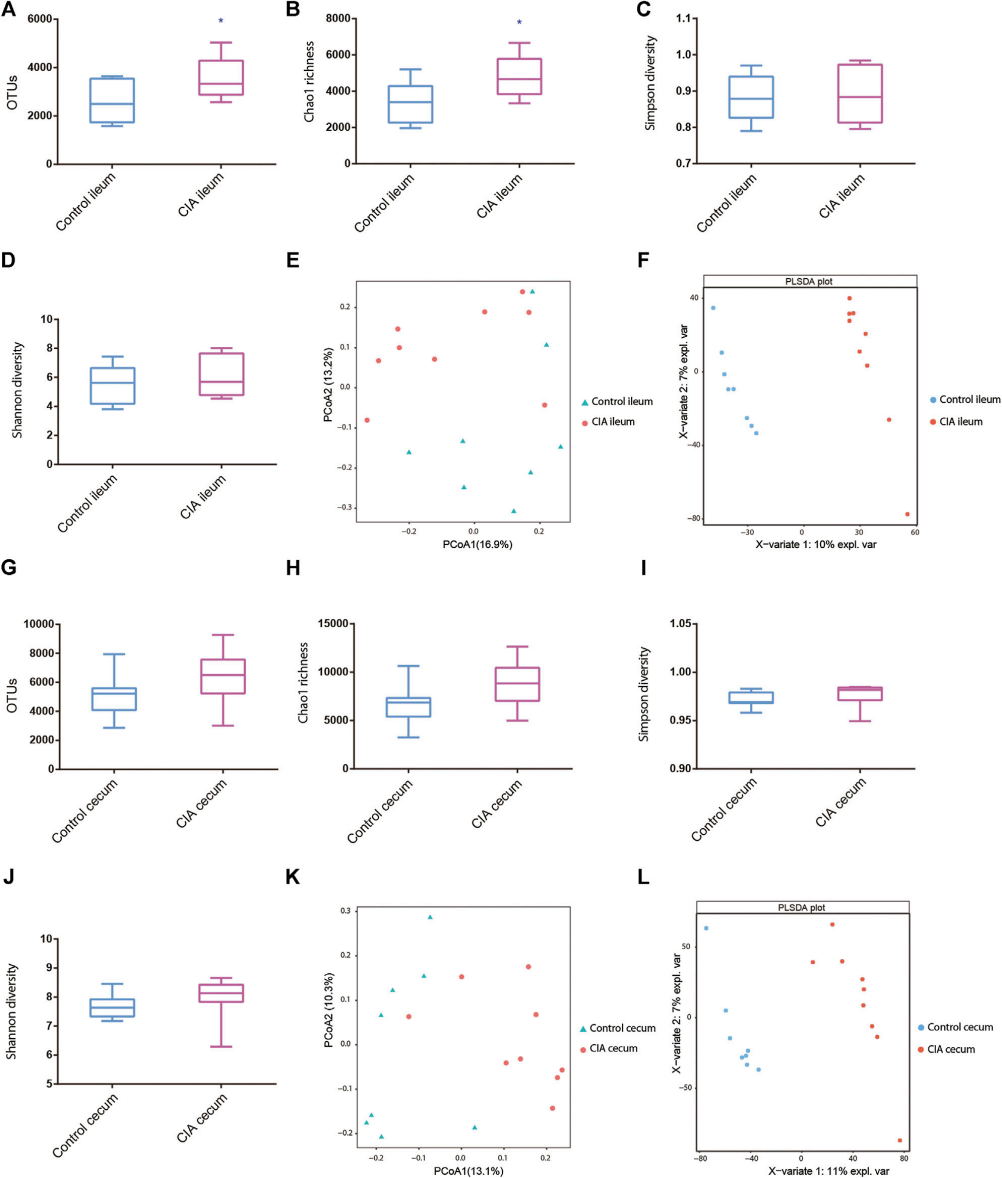
Ileal and cecal microbiota alterations in collagen-induced arthritis (CIA) rats compared with control rats based on the *α*- and *β*-diversity. **(A–D)** Alterations in the observed species, Chao richness estimator, Simpson diversity and Shannon diversity indices of the ileal microbial community. **(E,F)** Principal coordinate analysis (PCoA) and partial least squares discriminant analysis (PLSDA) of the ileum as determined by the unweighted UniFrac distance in CIA and control rats. **(G–J)** Alterations in the observed species, Chao richness estimator, Simpson diversity and Shannon diversity indices of the cecal microbial community. **(K,L)** PCoA and PLSDA of the cecum as determined by the unweighted UniFrac distance in CIA and control rats. The values are presented as the means ± SDs. *p < 0.05 compared with the control group.

In the ileum, the CIA rats exhibited significant increases in the observed species and Chao1 indices compared with those of the healthy rats (Figures 2A,B). However, in the cecum, although CIA rats also tended to exhibit increased OTUs and Chao1 values compared with those of the control rats, no significant difference was found between the two groups (Figures 2G,H). Additionally, no significant differences in the microbial diversities in the ileum and cecum were observed between the CIA and healthy rats, as demonstrated by the Shannon and Simpson indices (Figures 2C, D, I, J).

The PCoA results provided a visual separation of the microbial communities in the ileum between the CIA and control groups (Figure 2E), which indicated that CIA tended to affect the microbial community in the ileum of rats. Moreover, the PCoA results also revealed a visual separation of the microbial community in the cecum between the CIA and control groups (Figure 2K). However, the results were not significantly different between the ileum and cecum. Similar visual separation results were also obtained with partial least squares discriminant analysis (PLSDA) (Figures 2F,L).

### Collagen-Induced Arthritis Alters the Relative Abundances of the Microbiota at the Family Levels

**FIGURE 3:**
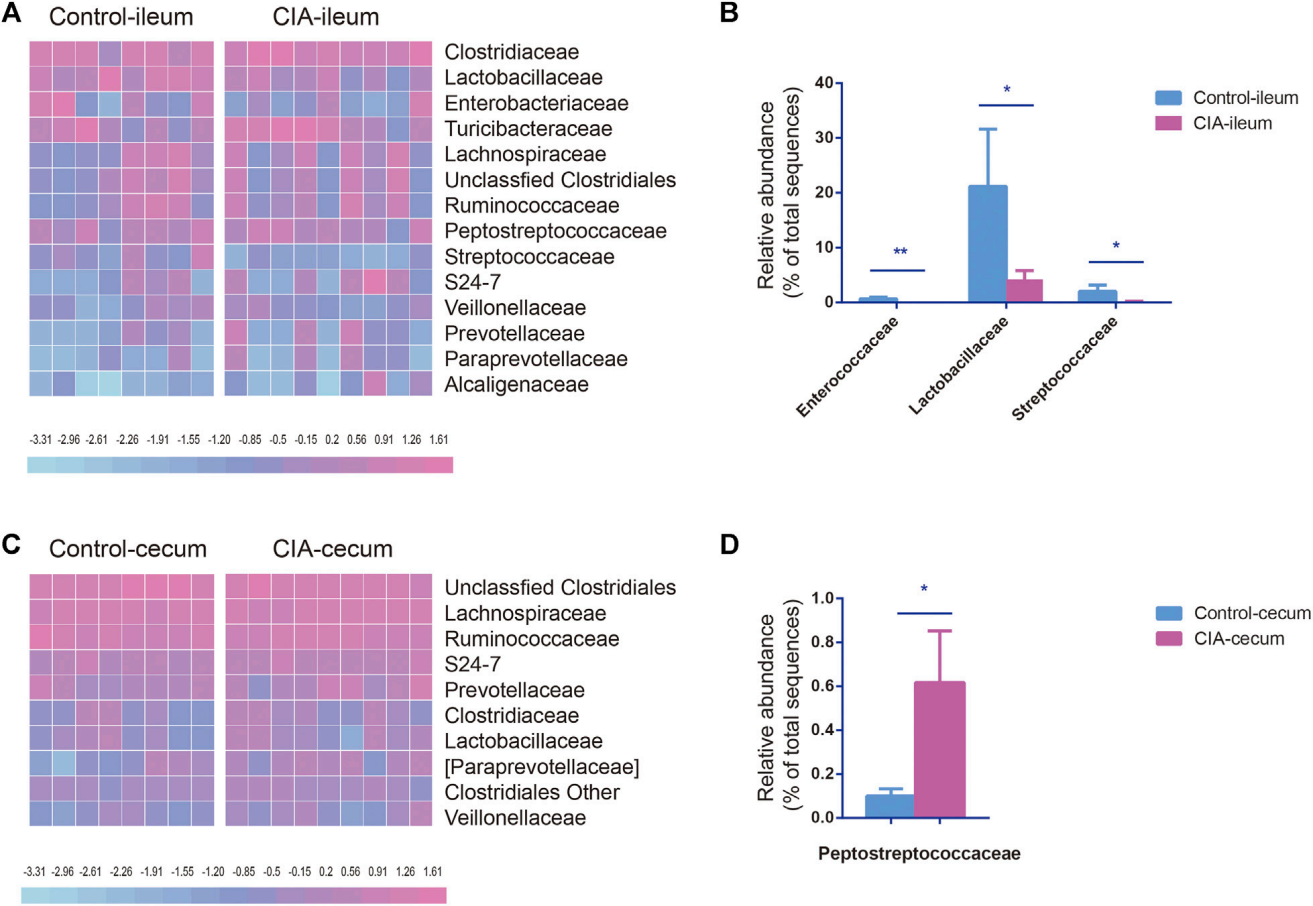
Comparison of the family-level compositions of the ileal and cecal microbiota communities in the control and collagen-induced arthritis (CIA) groups. **(A)** Heat map of the relative abundances at the family level. **(B)** Significantly changed microbial families found in the ileum. **(C)** Heat map of the relative abundances at the family level. **(D)** Significantly changed microbial families found in the cecum. The values are presented as the means ± SDs. *p < 0.05 and **p < 0.01 compared with the control group.

To account for the differences in microbial diversity, we further identified the bacteria at the family and genus levels. We selected families with a relative abundance >1% in at least one group, as shown in the heat maps presented in Figures 3A,C. The family-level analysis showed that the abundances of 16 and 19 families were significantly changed in the ileum (Supplementary Figure S3A) and cecum (Supplementary Figure S3B) of the CIA group, respectively. Among the families with a relative abundance greater than 0.5% in at least one group, Enterococcaceae (*p* < 0.01), Lactobacillaceae (*p* < 0.05) and Streptococcaceae (*p* < 0.05) presented significantly lower abundances in the ileum of the CIA group than in that of the control group (Figure 3B). However, in the cecum, the abundance of the bacterial family Peptostreptococcaceae (*p* < 0.05) was significantly increased after CIA induction (Figure 3D).

### Collagen-Induced Arthritis Alters the Relative Abundances of the Microbiota at the Genus Levels

**FIGURE 4:**
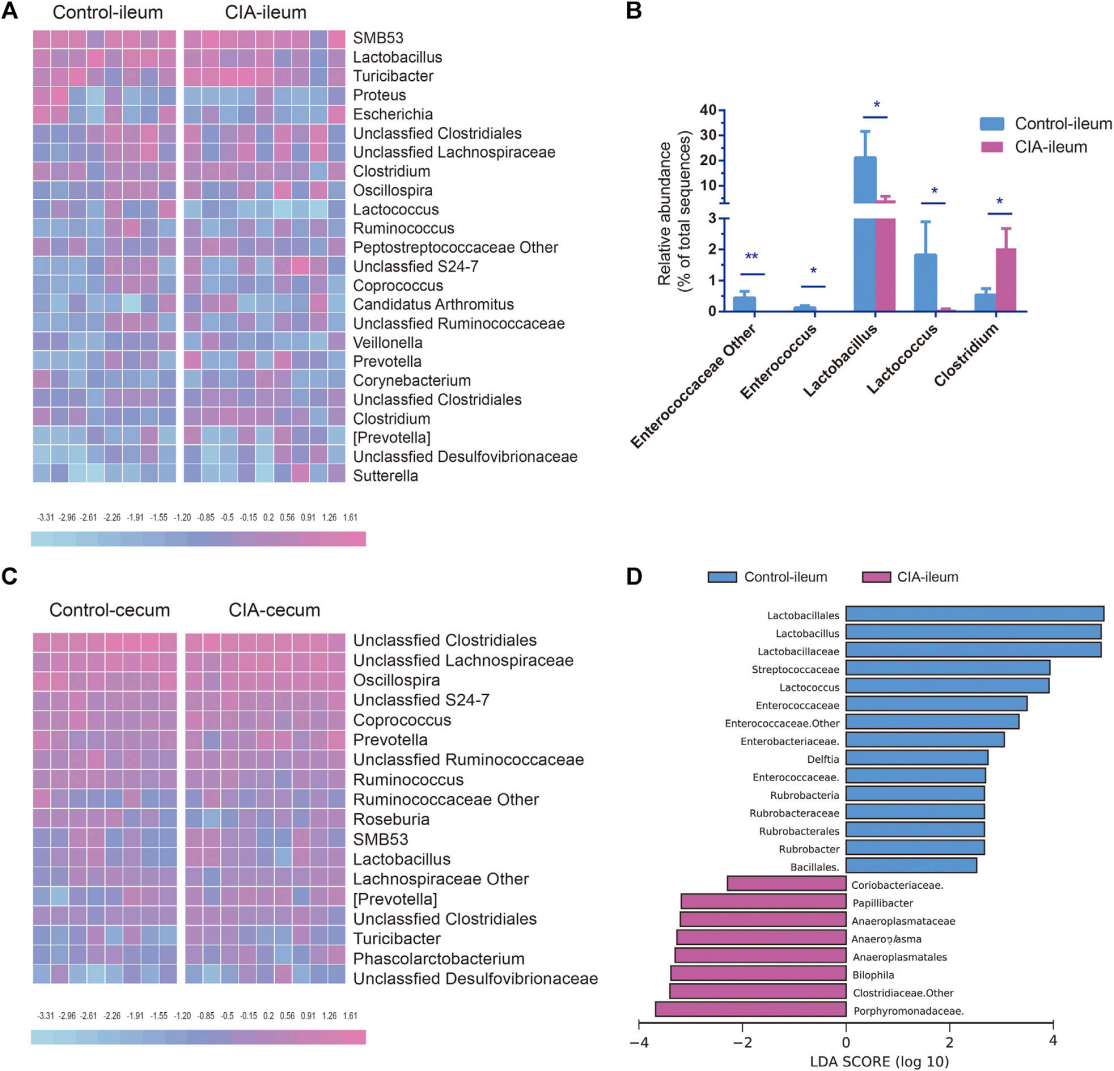
Comparison of the genus-level microbial communities in the ileum and cecum of the control and collagen-induced arthritis (CIA) groups and taxa-level changes in the ileal microbiota. **(A)** Heat map of the relative abundances at the genus level. **(B)** Significantly changed microbial genera found in ileal digesta. **(C)** Heat map of the relative abundances at the genus level. **(D)** The linear discriminant analysis effect size algorithm revealed taxonomic changes in the ileal microbiota. The bar plot shows the significantly altered taxa (*p* < 0.05) based on the effect size [linear discriminant analysis score (log_10_) > ±2). The values are presented as the means ± SDs. *p < 0.05 and **p < 0.01 compared with the control group.

The genus-level changes in genera with a relative abundance greater than 0.5% in at least one group are shown as a heat map in Figures 4A,C. At the genus level, the abundances of 28 and 30 genera were changed significantly in the ileum and cecum of the CIA group, respectively. CIA induction significantly reduced the abundances of *Lactobacillus*, *Lactococcus*, unclassified Enterococcaceae *Enterococcus* (*p* < 0.05) and other genera of Enterococcaceae (*p* < 0.01) in the ileum but increased the abundance of *Clostridium* in the ileum (*p* < 0.05, Figure 4B). *Lactobacillus*, *Lactococcus*, and *Clostridium* were the three most abundant genera in the ileum that exhibited significant changes in abundance after CIA induction (>1.5% in at least one group). Among these genera, *Lactobacillus* was the most abundant (21.15% in the control group and 10.48% in the CIA group). In the cecum, unclassified *Clostridiales*, unclassified Lachnospiraceae and *Oscillospira* were the three most abundant genera in both the control and CIA groups, but no significant differences in the abundances of these genera were found. Interestingly, all the genera that exhibited changes in the cecum presented an extremely low relative abundance (<0.5%). In summary, these results indicate that CIA induction significantly changed the gut microbiota communities, particularly in the ileum digesta. Thus, the LEfSe algorithm was then used to further identify specific bacterial taxa that are characteristic of the ileum of the CIA group but not in that of the control group. Consequently, the abundances of 23 taxa were found different between the CIA and control groups. The abundances of 15 taxa were increased in the control group, and those of eight taxa were increased in the CIA group (Figure 4D).

### The Relative mRNA and Protein Expression of Interleukin-1β, and Interleukin-17A Is Increased in the Ileum of the Collagen-Induced Arthritis Group

**FIGURE 5:**
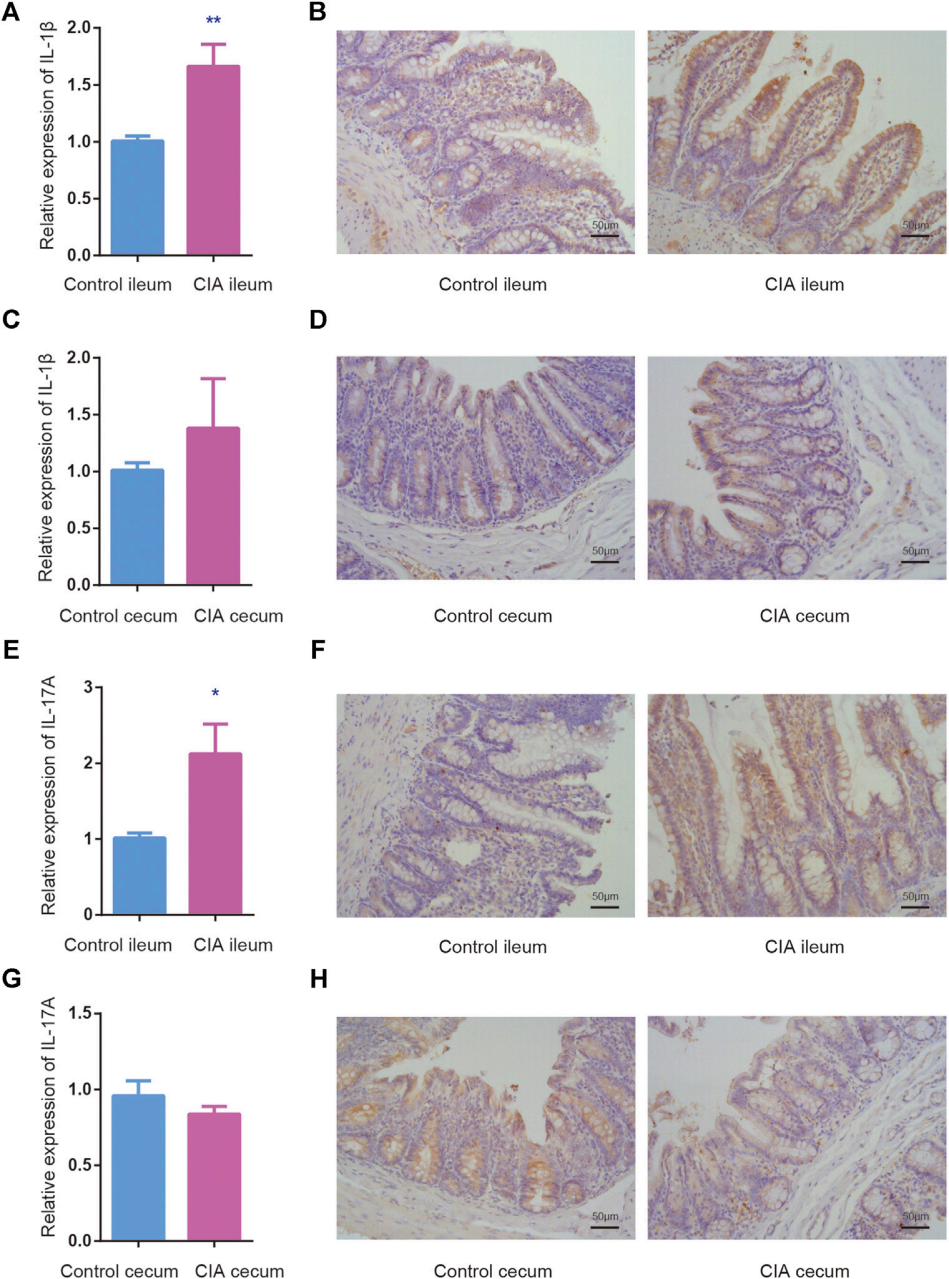
The relative mRNA and protein expression of interleukin (IL)-1β and IL-17A was upregulated in the mucosal layer of ileum of the collagen-induced arthritis (CIA) group. The mRNA expression was determined relative to the mean value of the control group. **(A,B)** The relative mRNA and protein expression of IL-1β in the mucosal layer of the ileum. **(C,D)** The relative mRNA and protein expression of IL-1β in the mucosal layer of the cecum. **(E,F)** The relative mRNA and protein expression of IL-17A in the mucosal layer of the ileum. **(G,H)** The relative mRNA and protein expression of IL-17A in the mucosal layer of the cecum. The data are presented as the means ± SEMs. *p < 0.05 and **p < 0.01 compared with the control group.

Proinflammatory cytokines and autoantibodies are involved in the upregulation of inflammatory responses. IL-1β and IL-17A are markers of inflammatory responses in the intestine. To evaluate whether mucosa-mediated inflammatory cytokine production differs between the ileum and cecum, we assessed the mRNA expression of IL-1β, and IL-17A in the ileum and cecum of the CIA and control groups by real-time PCR. Interestingly, significantly increased mRNA levels of IL-1β, and IL-17A were found in only the ileum after CIA induction (Figures 5A,E). No significant difference in IL-1β, and IL-17A mRNA expression in the cecum was found between CIA and control rats (Figures 5C,G). The protein expression in the epithelium and lamina propria of the mucosal layer was detected by immunohistochemistry as shown in Figure 5 (right panel).

## Discussion

RA is a systemic autoimmune disease that results in joint inflammation, cartilage damage and bone destruction, and researchers have become increasingly interested in studying the interplay of gut microbiota dysbiosis and the pathogenesis of RA (Maeda and Takeda, 2017). Microbial analyses of fecal samples have been used in a variety of studies to explore this relationship because these samples can be conveniently obtained and are thought to represent all gut microbial alterations of the host. However, the GIT is a multiorgan system, and each of its regions exhibits functional heterogeneity to regulate digestion, nutrient absorption, immunity and metabolism. These properties give rise to regional differences among the extensive gut microbial compositions in the diverse GIT segments of the host (Martinez-Guryn et al., 2019). A previous study revealed that in healthy conditions, the lower GIT is composed of distinct microbial populations along the small intestine, cecum and colon, and the small intestine exhibits lower microbial diversity than the cecum and colon (Donaldson et al., 2016). Differences in the gut microbiota between the ileum and cecum have been found in studies of diverse species. Through terminal restriction fragment length polymorphism (T-RFLP) and 16S rRNA sequence analyses, Gong et al. found that the microbial community in the ileum was less diverse than that in the cecum of broiler chickens (Gong et al., 2002). Yu et al. demonstrated that early antibiotic exposure exerts varying effects on the microbial communities in the ileum and cecum, and this treatment has more substantial effects in the ileum than in the cecum by altering the diversity, richness and structure of the genera (Yu et al., 2018). In a study of aged rats, Lee et al. revealed that the ß-diversity of the microbiota composition in the ileum was higher than that in the cecum, but the microbiota a-diversity was lower in the ileum than in the cecum. Lactobacillaceae were found at higher abundances in the ileum than in the cecum, whereas Ruminococcaceae and Lachnospiraceae were more abundant in the cecum (Lee et al., 2018). The microbial properties are affected by CIA induction, and the gut microbiota in different locations of the GIT exist in different physicochemical, nutrient and immune-related conditions. A previous study demonstrated that the changes induced by CIA in the gut microbial communities of mice differ between ileal and colonic microbes (Doonan et al., 2019). Interestingly, when transferred into germ-free mice, fecal ileal samples from only arthritic mice immunized with “arthritogenic” collagen type II + complete Freund’s adjuvant increased gut permeability, while those from the other regimen did not (Tajik et al., 2020). Therefore, the measurement of only fecal samples might not be sufficient for exploring the onset and development of CIA, which are mediated by the numerous gut microbiota in the host. Characterizing the microbial alterations in distinct intestinal compartments of CIA rats might provide more representative samples for exploring the pathogenesis of arthritis. In the present study, we compared the specific differences in the microbiota that colonize the ileum and cecum of CIA rats and detected the regulation of certain gut-associated immunologic factors.

The main functions of the large intestine are to extract water and salt from solid wastes, whereas the small intestine mainly functions to absorb nutrients and minerals from the diet. The ileum and cecum, which are part of the small and large intestines, respectively, contain microbial populations with different structures. Herein, 16S rRNA gene sequencing showed that the microbiota in the ileal digesta of healthy control rats was significantly less diverse than that in the cecum, as demonstrated by the Shannon and Simpson indices, and this finding appears to be consistent with that obtained in a previous study (Donaldson et al., 2016). In CIA rats, less bacterial diversity was also found in the ileum than in the cecum based on the Shannon and Simpson indices. A previous study indicated that the *α*-diversity of the gut microbiota in fecal samples of CIA rats did not differ from that of control rats (Shan et al., 2018). In this study, a comparison of the ileal microbiota of CIA rats with control rats revealed that CIA induction significantly increased the richness indices, but the diversity indices did not show significant differences. Furthermore, both the richness and diversity indices of the cecum samples of the CIA group were not significantly different from those in the control group. Consequently, the alterations in the ileal and cecal bacteria induced by CIA exhibited *a*-diversity-based differences. In addition, the structure of the gut microbiota might periodical change in the CIA rat model, as demonstrated by long-term experiments performed by Peng et al., (2019). Jeong Y et al. found that the phylum Bacteroidetes is enriched in early RA patients (Jeong et al., 2019), and Rogier et al. found that the abundance of the family Lactobacillaceae, which belongs to the Firmicutes family, is reduced during the preclinical phase of arthritis (Rogier et al., 2017). At the phylum level, three predominant bacteria in the ileum were herein altered in CIA rats compared with the control group. Firmicutes (81.49%), Proteobacteria (15.20%) and Bacteroidetes (2.41%) were the three major groups in the ileum of the healthy control group. After CIA induction, the three predominant bacterial phyla in the ileum changed to Firmicutes (77.99%), Bacteroidetes (13.93%), and Proteobacteria (6.99%). However, the three dominant bacterial phyla in the cecum of the CIA group did not differ from those in the control group. At the family level, the abundances of the families Enterococcaceae, Lactobacillaceae and Streptococcaceae were significantly reduced in the ileum of the CIA group compared with the control group ileum. In the cecum, the abundance of the family Peptostreptococcaceae was significantly increased after CIA induction.

At the genus level, CIA induction influenced the ileal microbiota by substantially decreasing the abundances of *Lactobacillus* and *Lactococcus.* Lactic acid bacteria constitute a representative intervention for reducing autoimmunity and thus recovering immune homeostasis. The genus *Lactobacillus*, which belongs to the family Lactobacteriaceae, is the predominant genus in the small intestine. Some strains of *Lactobacillus* might exert beneficial effects on the immunomodulation of the immune response during CIA progression. Esvaran et al. showed that the oral administration of *Lactobacillus* species, such as *L. fermentum* PC1, to CIA mice could significantly decrease joint inflammation by increasing the production of the cytokines IL-4 and IL-10 and decreasing IL-12 production (Esvaran and Conway, 2019). A previous study suggested that *L. acidophilus* can protect rats from arthritis symptoms (Amdekar and Singh, 2016). Hosoya et al. showed that *L. helveticus* SBT2171 suppresses the excessive proliferation of LPS-stimulated mouse T lymphocytes and B lymphocytes and exerts an immunosuppressive effect *in vivo*, which indicates a possible mechanism for the alleviation of CIA (Hosoya et al., 2014). Yamashita et al. demonstrated that both the oral administration and intraperitoneal inoculation of *L. helveticus* SBT2171 attenuated CIA-related inflammatory symptoms, and intraperitoneal inoculation even decreased the numbers of CIA-exacerbating immune cells, including B cells and CD4^+^ T cells, and downregulated the production of IL-6 and bovine type II collagen-specific antibodies in CIA mice (Yamashita et al., 2017). Liu et al. found that pretreatment of CIA mice with *L. salivarius* UCC118 or *L. plantarum* WCFS1 isolated from RA patients markedly reduced the Th17 cell fraction, increased the Treg fraction, and enhanced the antiarthritic and anti-inflammatory effects; the former even notably increased the serum levels of the anti-inflammatory cytokine IL-10 (Liu et al., 2016). The abundance of the genus *Lactococcus* in the ileum of the CIA group was also markedly lower than that in the control group. The oral administration of an adapted recombinant strain, *L. lactis*, which was engineered to express murine IL-35, inhibits IL-17 and IFN-γ and increases IL-10 production derived from CCR6^+^ and CCR6^−^ Foxp3^+ or −^ CD39^+^ CD4^+^ T cells to suppress the incidence and progression of CIA (Maddaloni et al., 2018). The feeding of arthritic mice with food-based *Lactococcus* engineered to express CFA/I fimbriae prevents arthritis by inducing CD39^+^ Tregs to secrete TGF-β and IL-10 and thus inhibit TNF-α production and neutrophil influx into the joints (Maddaloni et al., 2015). Although the relative abundances of ileal *Lactobacillus* and *Lactococcus* were significantly reduced in the CIA group, a significantly increased abundance of *Clostridium* was observed. Some species of Clostridia are commensal, whereas others are pathogenic. The abundance of *Clostridium* III is higher in RA cohorts than in healthy controls, as revealed in a comparative study (Forbes et al., 2018). Different intestinal *Clostridium* species (*C. perfringens*, *C. histolyticum*, *C. clostridioforme*, *C. leptum*, *C. sporosphaeroides* and *Blautia coccoides*) evoke distinct responses regarding the production of cytokines, including TNF-α, IL-8 and IL-10, by human mononuclear cells and might subsequently influence immune responses (Tuovinen et al., 2013).

Considerable evidence indicates that the gut microbiota regulates the mucosal immune system, including multiple types of immunocytes that contribute to the development of RA (Lucchino et al., 2019). The results from experimental animal studies indicate that gut microbiota dysbiosis serves as a potential trigger of enteric mucosal immune responses and can thus lead to the development of CIA (Jubair et al., 2018). Given the different changes in the bacterial load between the ileum and cecum of CIA rats, we investigated whether the mRNA expression of immunological factors, specifically IL-1β and IL-17A, in these two compartments was altered by CIA induction to broaden our understanding of the interplay between gut microbiota and immunity in CIA rats. Interestingly, the mRNA expression of IL-1β and IL-17A in the epithelium and lamina propria of the mucosal layer of the ileum and cecum also showed differential regulation of the enteric mucosal immune responses between the CIA and control groups. The published data have shown that CIA induction increases the levels of the proinflammatory cytokines IL-1β and IL-17A (Chen et al., 2019; Hui et al., 2019). IL-17A is the primary cytokine of Th17 cells (Maddur et al., 2012), and IL-1β can induce IL-17A secretion from CD4^+^ T cells, which have been implicated in the pathogenesis of RA (Yang et al., 2008). A recent study showed that partial elimination of the gut microbiota by broad-spectrum antibiotics during the establishment of CIA regulates the mucosal T helper cell balance and inhibits IL-17A mRNA in the terminal ileum, which is a main site for microbiota-induced T cell regulation, whereas the expression of IL-17 in colon tissue is not affected by CIA. These researchers also analyzed the percentage of local Th17 cells in joint-draining lymph nodes and found that it was markedly higher in CIA mice at the preclinical phase than in the control mice (Rogier et al., 2017). Dendritic cells (DCs) have the unique ability to polarize specific types of T cell responses due to the production of polarizing cytokines. Mann et al. suggested that ileal DCs play a more inflammatory role than colonic DCs because more ileal DCs produce the proinflammatory cytokine IL-1β than colonic DCs (Mann et al., 2016). Thus, we detected the expression of IL-1β and IL-17A in the ileal and cecal epithelium and lamina propria of the mucosal layer. Higher levels of IL-1β and IL-17A expression were found in the ileal epithelium and lamina propria of the mucosal layer, but no significant differences in their expression in the cecal epithelium and lamina propria of the mucosal layer were found between the CIA and healthy control groups. Because all the changed bacterial families and genera in the cecum presented extremely low relative abundances, differences in bacteria with an extremely low abundance in the cecum might not be responsible for the progression of CIA. However, the ileal microbiota was more markedly influenced, as demonstrated by sharp reductions in the abundances of the family Lactobacillaceae and the genera *Lactobacillus* after CIA. In addition, because the microbial profile of the cecal digesta sample was not closely correlated with the gut-associated immunologic factors IL-1β and IL-17A, the changes in the ileal bacteria might be more reflective of the impact on CIA development than the alterations in the cecal bacteria. However, further studies need to explore the different species in these genera that exhibit significant changes in the ileal digesta and the effects of their diverse metabolites. In addition, the neighboring intestinal immunomodulatory cells that located in distinct intestinal mucosa may be the most likely intermediary by which the gut microbiota can influence the occurrence and development of RA (Xu et al., 2020), so the mechanisms by which the dysbiosis of microbiota and their metabolites in distinct intestinal positions affect intestinal Th17 cells and other related immunocyte responses should be investigated.

## Conclusion

In summary, this work demonstrates that the different alterations in bacterial taxa in the ileum and cecum and the expression of the *Lactobacillus* 16S rRNA gene in the ileum exhibit the most marked changes after CIA. The features of the ileal microbial community identified by differential abundance analysis are consistent with the immune-mediated inflammatory features of CIA, which suggests that the ileal microbiota might better represent the CIA-induced inflammatory responses than the cecal microbiota. These findings might be partially related to the altered induction of excessive mucosal IL-1β and IL-17A expression in the ileum, which highlights the interactions between the microbiota and CIA. The alterations in the abundances of specific bacteria in the ileum of the host further emphasize the potential effects on CIA development. These taxa might serve as biomarkers for the detection and diagnosis of RA in the clinic and might also be common components of RA etiology in different tissues. The potential effects of these observed changes on the intestinal mucosal system in RA need to be further investigated in the future.

## Data Availability Statement

The datasets generated for this study can be found in NCBI accession PRJNA668272.

## Ethics Statement

The animal experimental protocols were approved by the Research Ethics Committee of the Institute of Clinical Medical Sciences, China-Japan Friendship Hospital (No. 180207).

## Author Contributions

HX, JC, and XYL contributed equally to this paper. HX drafted the manuscript. HX, JC, and XYL conducted the experiments. XCL analyzed the data. YX and DF participated in discussions related to the paper. CX formulated the concept and designed the paper. HZ and DJ revised this paper. All the authors read and approved the final manuscript.

## Funding

This study was supported by the National Natural Science Foundation of China [grant numbers 81673844 and 81573845] and the Fundamental Research Funds for the Central Public Welfare Research Institutes [grant number ZZ2018001].

## Conflict of Interest

The authors declare that the research was conducted in the absence of any commercial or financial relationships that could be construed as a potential conflict of interest.
