# Supplementary material for: Regional Differences in the Gut Microbiota and Gut-Associated Immunologic Factors in the Ileum and Cecum of Rats With Collagen-Induced Arthritis
Source: Front Pharmacol. 2020 Nov 24;11:587534. doi: 10.3389/fphar.2020.587534 (PMC7797777; doi:10.3389/fphar.2020.587534)
Supplement: Supplementary file 1 [file datasheet1.docx]

**Supplementary Materials**

**Regional Differences in the Gut Microbiota and Gut-associated Immunologic Factors in the Ileum and Cecum of Rats with Collagen-induced Arthritis**

**Huihui Xu**^1^**^#^,** **Jinfeng Cao^1#^, Xiaoya Li^2,3#^, Xiangchen Lu^4,2^, Ya Xia^4,2^, Danping Fan^2,3^, Hongyan Zhao^1^*****, Dahong Ju^1^*****, Cheng Xiao^2,3,5^***

*Correspondence: Hongyan Zhao, zhaohongyan1997@163.com; Dahong Ju, judahong@126.com; Cheng Xiao, Cheng Xiao, xc2002812@126.com.

**1. Incidence and arthritis scores**

The arthritis scores of the CIA group increased significantly after immunization (*P*<0.01*,* Supplementary Figure 1A and B), and this group also experienced joint destruction, as detected by micro-CT (Supplementary Figure 1C). The ankle pathology of the CIA rats showed increased inflammatory cell infiltration and synovial proliferation compared with that in the control group (Supplementary Figure 1D).


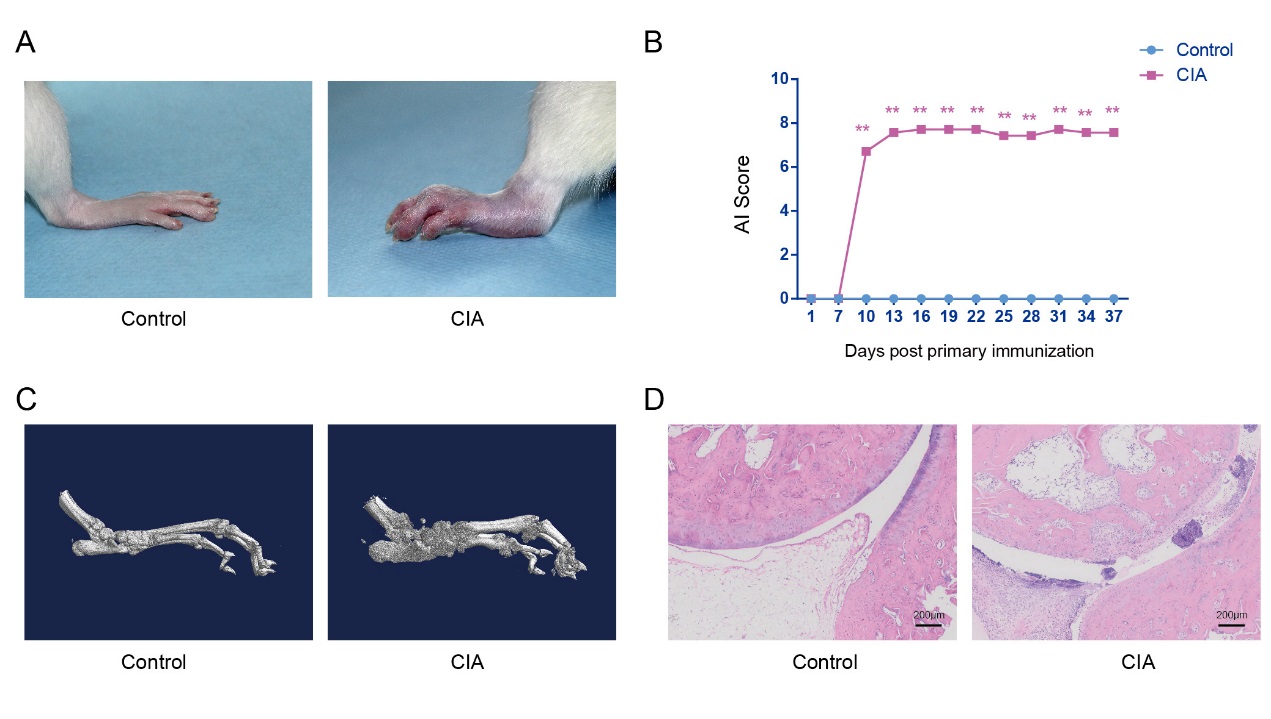


*Supplementary Figure 1. Comparison of arthritic inflammation and bone destruction in the control and CIA groups 38 days after the primary immunization. (A) Ankle joints of control and CIA rats. (B) The line plots represent the AI of the CIA rats. (C) Representative three-dimensional renderings of the ankle joint by micro-CT. (D) Representative pathological sections of the ankle joint by hematoxylin and eosin (H&E) staining. **P*<0.01 *compared with the other group.*

**2. Normal rats exhibit different microbial diversities and community structures in the ileum and cecum**

On average, 199,176±36,159 high-quality sequence reads from each sample were used for subsequent analysis by high-throughput sequencing of bacterial 16S rRNA. Ambiguous base sequences were removed and merged into a complete read. The chimeras were identified and removed, and the remaining high-quality sequences were analyzed. A total of 23,726 OTUs were clustered with 97% sequence similarity between reads and assigned taxonomic lineages by comparison with the Greengenes 16S rDNA database.

No significant differences in the OTUs or Chao1 richness estimators were found between the ileum and cecum in the control groups (Supplementary Figures 2A and 2B). However, the control rats displayed statistically significant differences in microbial diversity between the ileum and cecum, as determined by the Shannon and Simpson indices (Supplementary Figures 2C and 2D). As shown in Supplementary Figure 2E, the PCoA analysis results indicated a clear separation between the ileal and cecal microbial communities based on β-diversity, and the divergence in the distribution of the microbiota was statistically significant (ANOSIM, R=0.768, *P<*0.01). Additionally, a similar visual separation was achieved by NMDS ordination (Supplementary Figure 2F). Overall, both the ileum and cecum of healthy rats showed obvious differences in bacterial diversity. The abundant phyla (≥0.05% in one group) are listed in Supplementary Figure 2G. The phylum-level analysis revealed that Firmicutes (81.49%), Proteobacteria (15.20%), and Bacteroidetes (2.41%) were the three predominant phyla in the ileum of control rats, and these represented more than 99% of the total sequences. In addition, based on their relative abundances, Firmicutes (89.03%) and Bacteroidetes (10.35%) were the two predominant phyla in the cecum of the healthy rats and together accounted for more than 99% of the total sequences. The abundances of Actinobacteria (*P<*0.01), Bacteroidetes (*P<*0.01) and Proteobacteria (*P<*0.05) showed significant differences between the ileum and cecum of healthy rats (Supplementary Figure 2H). Among these bacteria, the relative abundances of Actinobacteria and Proteobacteria were markedly higher in the ileum than in the cecum, but the relative abundance of Bacteroidetes was significantly lower in the ileum than in the cecum.


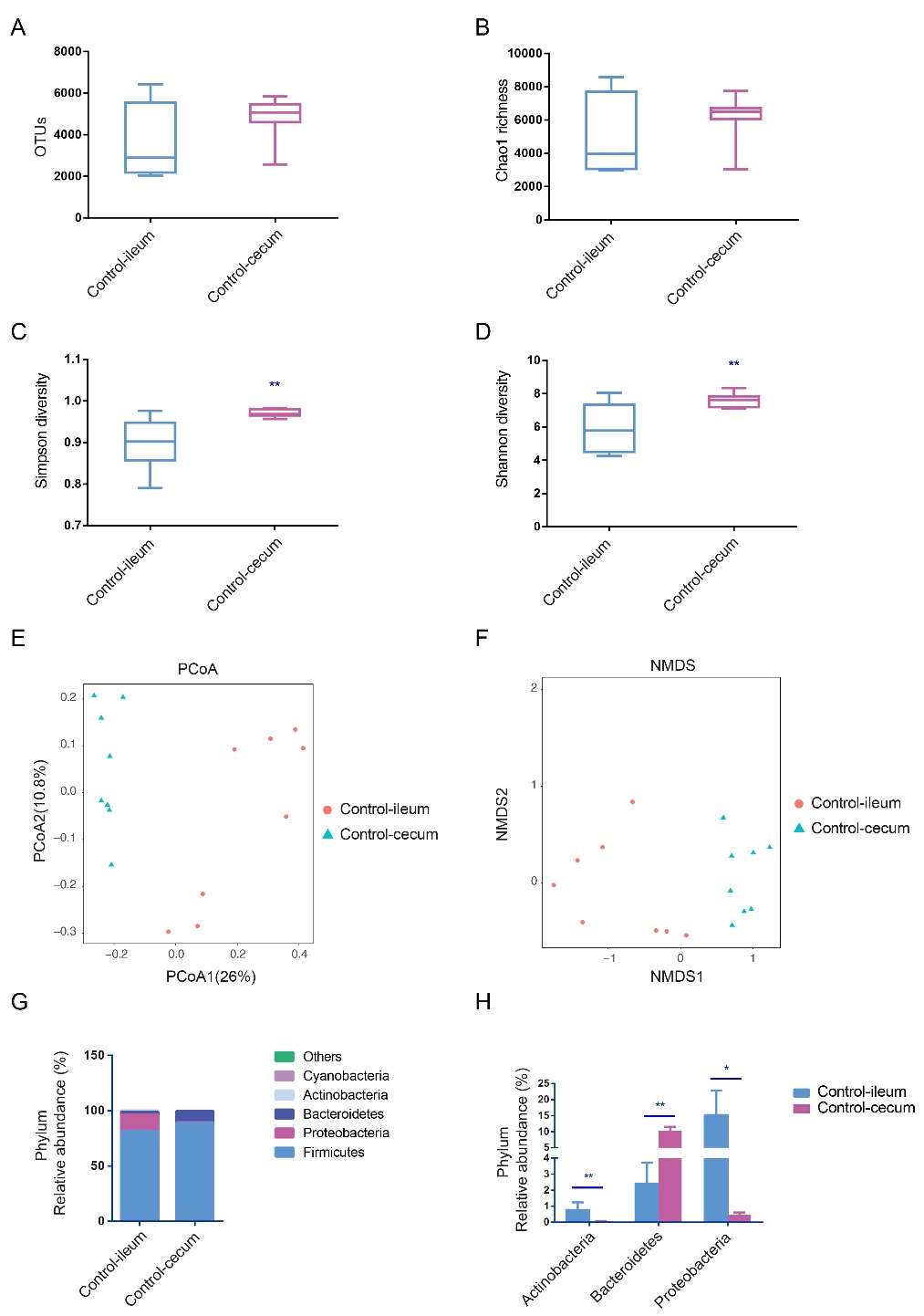


*Supplementary Figure 2. The ileal and cecal microbial communities in the control group were obtained by 16S sequencing. (A-B) Analysis of the observed species and Chao richness estimators in the ileal and cecal microbial communities. (C-D) Analysis of the Simpson diversity and Shannon diversity indices of the ileal and cecal microbial communities. (E) Community structure assessed by PCoA. (F) NMDS ordination of the microbiome. (G) Phylum-level taxonomic composition shown as the average relative abundance. (H) Significantly different microbial phyla found in the ileum and cecum. The values are presented as the means ± SDs. *P<0.05 and **P<0.01 compared with the ileum.*

3. **CIA alters the relative abundances of the microbiota at the family levels**

To account for the differences in microbial diversity, we further identified the bacteria at the family and genus levels. We selected families with a relative abundance >1% in at least one group, as shown in the heat maps presented in Figures 3A and 3C. The family-level analysis showed that the abundances of 16 and 19 families were significantly changed in the ileum (Supplementary Figure 3A) and cecum (Supplementary Figure 3B) of the CIA group, respectively.


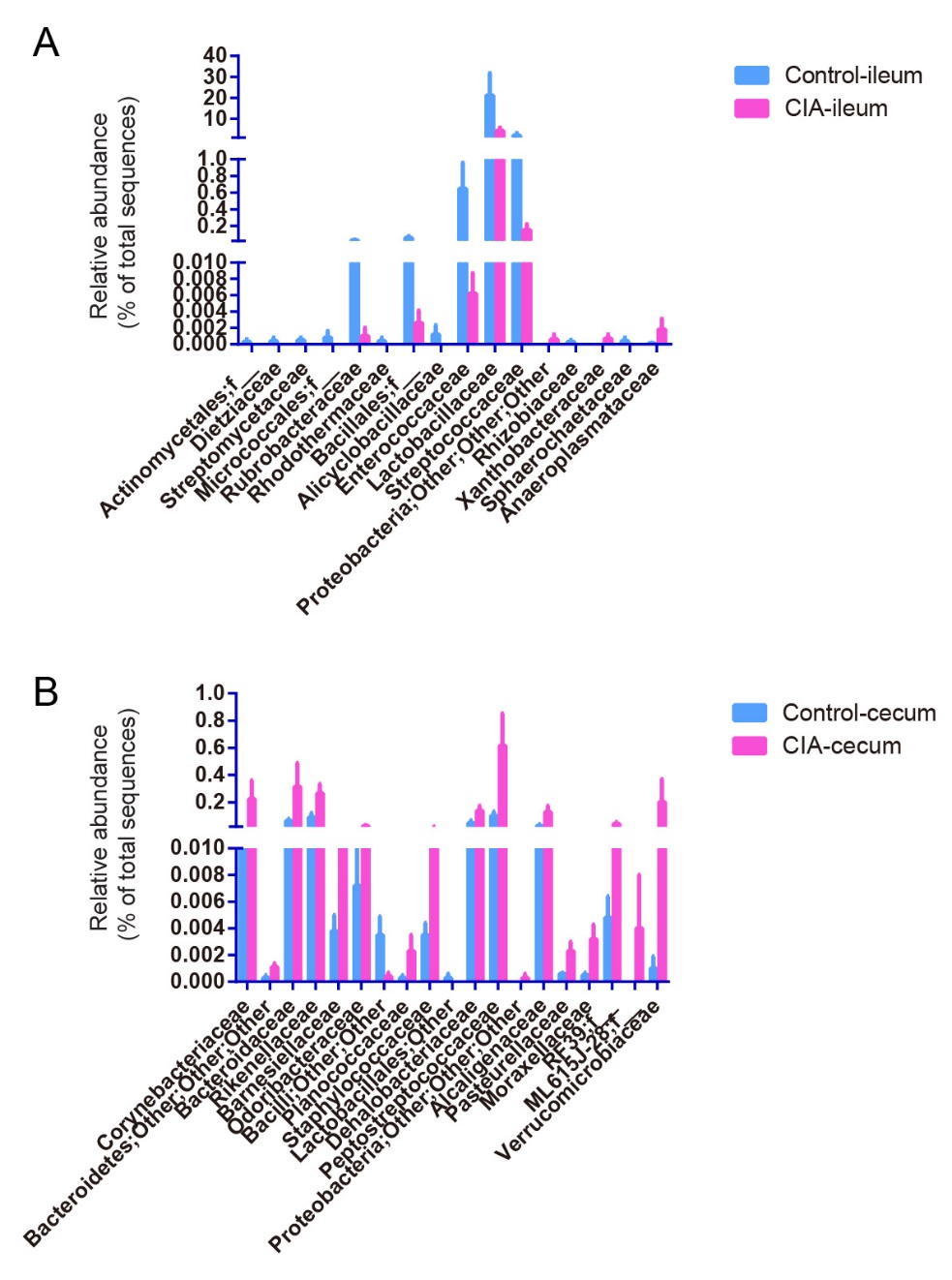


*Supplementary Figure 3.The significantly changed family in ileal (A) and cecal (B) digesta after CIA.*

**4. The** protein **expression of IL-1β, and IL-17A is increased in the ileum of the CIA group by immunohistochemistry.**

Partial tissue samples of the ileum and cecum were harvested after the contents were collected, flushed with physiological saline, dissected longitudinally and fixed in formaldehyde. The tissues were subsequently embedded in paraffin, and sections of 4 μm thickness were cut from the paraffin-embedded tissues. SIgA, IL-1β, and IL-17A were immunolocalized in tissues according to the manufacturer’s instructions. The paraffin sections were dewaxed using routine methods, incubated with 3% H_2_O_2_ for 10 min and treated with primary antibodies against rat SIgA (Abcam, Cambridge, MA, United States), IL-1β (Santa Cruz Biotechnology) and IL-17A (Abcam) at 37°C for 2 h. The sections were then incubated with poly-HRP anti-rabbit or anti-goat IgG for 10 min at room temperature, stained with 3,3-diaminobenzidine (Fuzhou Maixin Biotech. Co., Ltd.) and counterstained with hematoxylin.
